# Electroacupuncture Improves Blood Pressure in SHRs by Regulating the Immune Balance between Th17 and Treg

**DOI:** 10.1155/2020/5375981

**Published:** 2020-07-07

**Authors:** Yang Wang, Lili Zhang, Li Li, Hantong Hu, Pan Pan, Baoyu Zhang, Wenhua Ning, Mengxiong Zhao, Shu Wang

**Affiliations:** ^1^First Teaching Hospital of Tianjin University of Traditional Chinese Medicine, Tianjin 300193, China; ^2^Tianjin University of Traditional Chinese Medicine, Tianjin 300193, China; ^3^Tianjin Key Laboratory of Translational Research of TCM Prescription and Syndrome, Tianjin 300193, China; ^4^National Clinical Research Center for Chinese Medicine Acupuncture and Moxibustion, Tianjin 300193, China; ^5^Tianjin Key Laboratory of Acupuncture and Moxibustion, Tianjin 300193, China; ^6^The Third Affiliated Hospital of Zhejiang Chinese Medical University, Hangzhou 313202, China

## Abstract

The present study investigated the effects of electroacupuncture on blood pressure in spontaneously hypertensive rats (SHRs) by regulating the immune balance of T helper 17 cells (Th17 cells) and regulatory T cells (Treg cells). This study investigated the role of electroacupuncture in the immune balance of SHRs using Western blot, flow cytometry, and ELISA techniques. Electroacupuncture significantly improved blood pressure, downregulated the expression of ROR*γ*t, and upregulated the expression of Foxp3, reduced the production of Th17 cells, promoted the production of Treg cells, reduced the secretion of IL-6 and IL-17, and increased the secretion of TGF-*β*1 and IL-10. These findings suggest that electroacupuncture therapy effectively improved the systolic blood pressure of SHRs, and its mechanism may be related to promotion of the immune balance between Th17 and Treg.

## 1. Introduction

Hypertension is a major public health problem worldwide and an independent risk factor for the heart, brain, kidney, and blood vessel diseases [1]. Inflammation and autoimmunity play key roles in the occurrence and development of hypertension [2]. Previous studies have found that the intensity of the inflammatory cells infiltration positively correlated with elevated blood pressure, and the infiltration of immune cells in the kidney may be one cause hypertension [3]. Therefore, improving autoimmunity and inflammation may be essential for the treatment of hypertension.

Immune dysregulation primarily manifests as dysregulation of the innate immune and adaptive immune systems. The adaptive immune system primarily includes inflammatory response factors released by the T cells, and different lymphocytes and the corresponding cytokines released by the T cells contribute to the damage of the targeted organs. There are four different phenotypes of CD4+ T cells: T helper 1 cells (Th1 cells), Th2 cells, Th17 cells, and the regulatory T (Treg) cells [2]. Previous studies on the imbalance between hypertension and Th cell subsets focused on Th1/Th2 [4], but Th17/Treg is increasingly valued in the study of hypertension and its target organ damage [5]. Th17 cells cause a highly inflammatory immune response, and the Treg cells play a key role in immune homeostasis. These two cell types play opposite roles in inflammation and immune response [6]. Under normal conditions, the effects of Treg cells and Th17 cells are in an equilibrium state, and the imbalance of Th17/Treg cells is at the heart of the pathogenesis of various diseases and conditions [7]. Therefore, the present study analysed Th17 cells and Treg cells.

The orphan nuclear receptor *γ*t (ROR*γ*t) is a key transcription factor that regulates the differentiation of the Th17 cells [8]. The Th17 cells secrete interleukin- (IL-) 6, IL-17 (also known as IL-17A), and other proinflammatory factors [9]. Treg cells are a subset of CD4+ cells that are characterized by the forkhead box P3 (Foxp3) and CD25 expression and play a key role in autoimmune tolerance [10]. Treg cells primarily secrete transforming growth factor-beta (TGF-*β*) and IL-10, which play an anti-inflammatory role [11, 12]. There are three main subtypes of TGF-*β* in mammals, TGF-*β*1, TGF-*β*2, and TGF-*β*3. TGF-*β*1 exhibits the main biological activity [13]. Th17 cells mediate the inflammatory responses, and the Treg cells mediate the immune tolerance. Therefore, the Th17/Treg immunobalance axis is more closely related to hypertension [3].

Acupuncture has become increasingly accepted as an alternative medicine in Western countries [14]. Acupuncture is an effective method to treat cardiovascular disease and, previous meta-analyses based on evidence-based medical demonstrated that it effectively reduced blood pressure [15, 16]. A meta-analysis [15] has confirmed that ST9 (*Renying*) and LR3 (*Taichong*) are two of the most widely used acupoints for the treatment of hypertension in clinical practice. Electroacupuncture is a modified form of acupuncture that combines traditional acupuncture with modern electrotherapy [17]. Some research showed that electroacupuncture produced a sympathoinhibitory effect that resulted in decreased cardiac sympathetic drive, vasodilation, and reduced blood pressure [18]. Previous studies showed that electroacupuncture had anti-inflammatory effects [19] and regulated the balance of Th17/Treg [20]. However, little is known about the mechanisms of electroacupuncture in the treatment of hypertension by regulating the immune balance between the Th17 and Treg. Therefore, we asked whether the hypotensive effect of electroacupuncture was related to the regulation of Th17/Treg and their inflammatory factors. Based on the pathogenesis of hypertension and the effects of electroacupuncture, we used spontaneously hypertensive rats (SHRs) as the research subjects to examine the role of electroacupuncture in improving the systolic blood pressure (SBP) by regulating the balance of Th17/Treg.

## 2. Materials and Methods

### 2.1. Animals

Twenty-four healthy 14-week-old male SHRs and eight healthy 14-week-old male Wistar-Kyoto (WKY) rats were purchased from Charles River (Animal production license SCXK(Jing) 2016–0006). The rats had free access to food and water under a 12 h light/dark cycle at 24 ± 2°C and 50 ± 10% of humidity. The experimental procedure was performed in accordance with the National Institute of Health Guidelines for Care and Use of Laboratory Animals (NIH Publication No. 8023, revised in 1985). The research protocol was approved by the Ethics Committee of the Institute of Radiation Medicine, Chinese Academy of Medical Sciences and Peking Union Medical College (approval number: IRM-DWLL-2018074).

### 2.2. Electroacupuncture and Drugs Treatment

**Figure 1:**
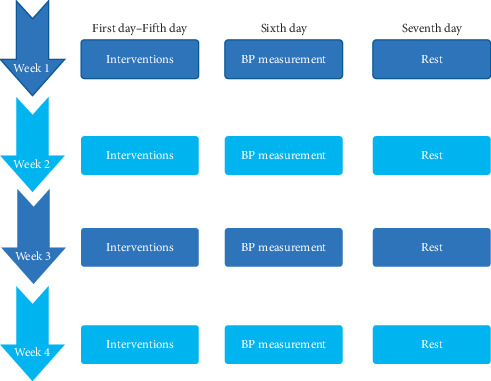
The flowchart of intervention procedure.

All rats were fed adaptively for three days, and their baseline SBP was then measured. Using a randomized block design, 24 SHRs were divided into three groups: electroacupuncture group (SHR + EA), antagonist group (SHR + AG490), and model control group (Model). Eight WKY rats were selected as the normal control group (Normal). Four rats were housed in each cage. For the SHR + EA, the bilateral ST9 (straight puncture 3 mm) and bilateral LR3 (straight puncture 2-3 mm) were selected (Table 1). The localization of these acupoints was based on the Laboratory Acupuncture and Atlas of Animal Acupoints enacted by the Experimental Acupuncture-Moxibustion Research Association of China Academy of Acupuncture and Moxibustion [21]. Disposable stainless-steel needles (0.25 ∗ 40 mm, Suzhou Medical Supplies Factory Co., Ltd.) were inserted into the ST9 and LR3, and the needle handles were connected to the electroacupuncture apparatus (HANS-100A, Nanjing Jisheng Medical Technology Co., Ltd.). The electrical stimulation parameters applied were 2/15 Hz and 1 mA, each lasting for 15 min, once daily, for 5 consecutive days (Figure 1). During the experimental process, the rats were kept awake and wrapped in special cloth to avoid any movement, struggling, or other stress responses. Rats in the SHR + AG490 received an intraperitoneal injection of 5 *μ*g/g AG490 (provided by Sigma) once daily for five consecutive days over four weeks. AG490 is an inhibitor of Janus kinase 2 that is widely used in various inflammatory diseases [22–25]. A previous study [22] showed that in vivo AG490 administration was safe in animals. In the Model and Normal, the same intensity of catching-grasping stimulation was applied once daily, for five consecutive days over four weeks. Blood pressure was measured on the sixth day, and the animals were rested on the seventh day during the four-week intervention.

### 2.3. BP Measurements

A rat tail noninvasive blood pressure meter (NIBP) (BP-98A; Softron, Japan), which is frequently used in similar studies [26], was used to measure the blood pressure and examine the effectiveness of the electroacupuncture on SBP in rats before intervention and after the weekly intervention.

### 2.4. Euthanasia and Blood Sampling

After treatment and blood pressure measurement, all rats were anaesthetized with sodium pentobarbital (50 mg/kg) via an intraperitoneal injection, and blood was collected via cardiac puncture. Some blood was extracted into heparin anticoagulant tubes for flow cytometry. Some blood was collected and allowed to clot for 2 hours at room temperature. This blood was centrifuged (1000 g for 15 min, 4°C), and the collected sera were analyzed using ELISA. After blood collection, the rats were euthanized via cervical dislocation.

### 2.5. Western Blot Assay

After cervical dislocation, the kidney was removed and immediately placed in a freezer at−80°C for later use. Western blot was used to detect the protein expression of Foxp3 and ROR*γ*t in the kidney. Kidney tissue 30–50 mg was crushed, and 500 *μ*l of lysate was added to extract the tissue protein. After centrifugation for 10 min at 12000 g, protein concentration in the supernatant was measured using the BCA protein assay. The supernatant was mixed with a loading buffer and boiled for 10 mins. After electrophoresis, proteins were transferred to a transmembrane, and the membranes were incubated with antibodies against Foxp3 and ROR*γ*t as the primary antibody and a secondary antibody (Table 2). The ECL chemiluminescence method was used to Visualize the blot. Images of the protein bands were taken, and the gray values were read using ImageJ. The target synaptophysin/actin ratio was calculated to show the expression of the related proteins.

### 2.6. Flow Cytometric Analysis

We used flow cytometry to verify the proliferation and differentiation of the Th17 cells and Treg cells in the blood. Blood samples were drawn into heparinized tubes and immediately used in the experiment. CD3+CD4+CD8a—IL-17+Th17 cells: cells were stimulated for four hours with Leuko Actvtn Cktl with Glgplg (BD Pharmingen) and RPMI Medium Modified (HyClone) in a constant temperature box at 37°C. Following the instructions of the IntraSureTMKit (BD Pharmingen), RBC Lysis Buffer (CWBIO) was added, and intracellular Foxp3 was stained. CD4+CD25+Foxp3+Treg cells: RBC Lysis Buffer (CWBIO) was added to the blood and washed with PBS, and the surface markers were stained with the corresponding fluorescent-labeled antibodies for 20 min. Intracellular staining of IL-17 was performed using the Transcription Factor Buffer Set (BD Pharmingen). The following antibodies were purchased from BD Pharmingen or Biolegend: anti-CD25-PE, anti-Foxp3-APC, anti-CD4-AF488, anti-CD3-APC, anti-CD8a-PerCP, and anti-IL-17-PE. Flow cytometric data were obtained using a FACSCalibur Flow Cytometer system (BD Pharmingen) and analyzed using FlowJo software.

### 2.7. Enzyme-Linked Immunosorbent Assay (ELISA)

The amounts of cytokines TGF-*β*1, IL-6, IL-10, and IL-17 in the collected sera were measured using sandwich ELISA. A rat TGF-*β*1, IL-6, IL-10 ELISA kit (BOSTER) and rat IL-17A ELISA kit (DAKEWE) were used according to the manufacturer's instructions to determine the TGF-*β*1, IL-6, IL-10, and IL-17 content in the serum. The absorbance at 450 nm was measured by using an ELISA microplate reader.

Please refer to the Supplementary Materials for more information about the reagent supplier.

### 2.8. Data Analysis

All data were analyzed using SPSS 22 and are expressed as means ± standard deviation (SD). Repeated measurements with two-way ANOVA were used to analyze the blood pressure data. Data obtained from multiple groups were compared using one-way ANOVA, and the LSD test was used to compare the data of two groups. *P* < 0.05 was considered statistically significant.

## 3. Results

### 3.1. The Effects of Electroacupuncture on SBP in Rats

**Figure 2:**
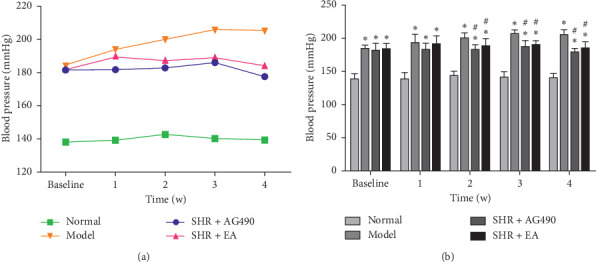
The SBP of rats in each group was measured using NIBP. (a) Trend chart of SBP in each group. (b) The SBP level of rats changed during the treatment time. Data are expressed as the means ± SD values. ^*∗*^*P* < 0.05, compared with Normal; ^#^*P* < 0.05, compared with Model.

There were no significant differences in baseline SBP between the Model, SHR + AG490, and SHR + EA before intervention (*P* > 0.05), which suggests that the three groups were comparable. The baseline SBP of SHRs was significantly higher than that of WKY (*P* < 0.05), and the baseline SBP of WKY was normal. The results of SBP of the rats after intervention showed a significant difference between groups (*P* < 0.05). When compared with the Normal, the SBP in the Model, SHR + AG490, and SHR + EA increased significantly (*P* < 0.05), which confirmed that the SHRs were hypertensive. When compared with the Model, the SBP of SHR + AG490 and SHR + EA decreased significantly after the second week of intervention (*P* < 0.05) (Table 3; Figure 2). The results showed that electroacupuncture significantly improved the SBP of SHRs.

### 3.2. Electroacupuncture Regulates Protein Expression in the Kidney

**Figure 3:**
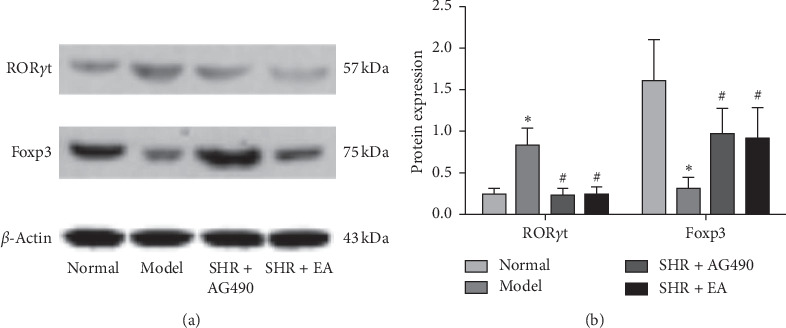
Protein expression in the kidney tissue of rats in each group. Western blot was used to analyze the expression of ROR*γ*t and Foxp3. (a) Protein expression and (b) protein expression analysis, *n* = 3. Data are expressed as the means ± SD values. ^*∗*^*P* < 0.05, compared with Normal; ^#^*P* < 0.05, compared with Model.

The expression of the ROR*γ*t protein in the Model increased significantly compared with the Normal (*P* < 0.05). When compared with the Model, the expression of ROR*γ*t in the SHR + AG490 and SHR + EA decreased significantly (*P* < 0.05). When compared with the Normal, the expression of Foxp3 protein in the Model decreased significantly (*P* < 0.05). When compared with the Model, the expression of Foxp3 in the SHR + AG490 and SHR + EA was upregulated (*P* < 0.05) (Figure 3). These results showed that electroacupuncture downregulated the expression of ROR*γ*t protein and upregulated Foxp3 protein expression.

### 3.3. Electroacupuncture Regulates Cell Proliferation and Differentiation

**Figure 4:**
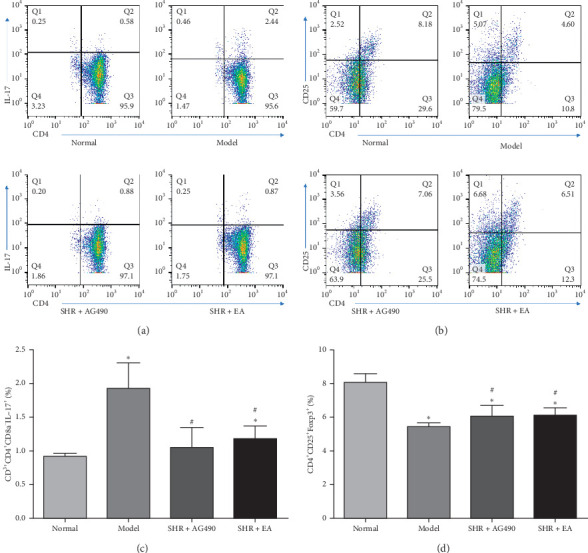
Flow cytometry was used to detect the proliferation and differentiation of Th17 and Treg cells in the blood. (a) Positive expression of Th17 cells. (b) Positive expression of Treg cells. (c) Proportion of Th17 cells, *n* = 8. (d) Proportion of Treg cells, *n* = 8. Data are expressed as the means ± SD values. ^*∗*^*P* < 0.05, compared with Normal; ^#^*P* < 0.05, compared with Model.

The number of Th17 cells in the Model and SHR+EA increased significantly compare with the Normal (*P* < 0.05). The number of Th17 cells in the SHR+AG490 and SHR+EA decreased significantly compared with the Model (*P* < 0.05). The number of Treg cells in the Model, SHR+AG490 and SHR+EA decreased significantly compared with the Normal (*P* < 0.05). The number of Treg cells in the SHR+AG490 and SHR+EA increased significantly compared with the Model (*P* < 0.05) (Figure 4). Therefore, electroacupuncture inhibited the proliferation and differentiation of Th17 cells and promoted the proliferation and differentiation of Treg cells.

### 3.4. Electroacupuncture Regulates the Secretion of Cytokines

**Figure 5:**
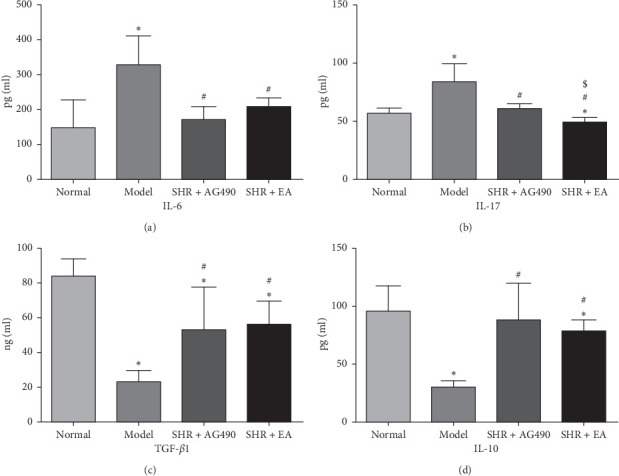
The concentrations of cytokines in rats. (a) The concentration of IL-6, *n* = 8. (b) The concentration of IL-17, *n* = 8. (c) The concentration of TGF-*β*1, *n* = 8. (d) The concentration of IL-10, *n* = 8. Data are expressed as the means ± SD values. ^*∗*^*P* < 0.05, compared with Normal; ^#^*P* < 0.05, compared with Model; ^$^*P* < 0.05, compared with SHR + AG490.

The concentration of IL-6 in the Model increased significantly compared with the Normal (*P* < 0.05). The concentration of IL-6 in the SHR+AG490 and SHR+EA decreased significantly compared with the Model (*P* < 0.05). The concentration of IL-17 in the Model increased significantly compared with the Normal (*P* < 0.05) but decreased significantly in the SHR+EA (*P* < 0.05). The concentration of IL-17 in the SHR+AG490 and SHR+EA decreased significantly compared with the Model (*P* < 0.05). The concentration of IL-17 in the SHR + EA was significantly lower than the SHR + AG490 (*P* < 0.05). The concentration of TGF-*β*1 in the Model, SHR+AG490, and SHR+EA decreased significantly compared with the Normal (*P* < 0.05). The concentration of TGF-*β*1 in the SHR+AG490 and SHR+EA increased significantly compared with that in the Model (*P* < 0.05). The concentration of IL-10 in the Model and SHR+EA decreased significantly compared with the Normal (*P* < 0.05). The concentration of IL-10 in the SHR+AG490 and SHR+EA increased significantly compared with the Model (*P* < 0.05) (Figure 5).

## 4. Discussion

The Th17 cells are a newly discovered T cell subset that play a key role in autoimmune diseases [3]. ROR*γ*t is a key transcription factor that regulates the differentiation of Th17 cells, and it is a therapeutic target for inflammatory diseases [8]. Another subset of T lymphocytes, Treg cells, inhibit innate and adaptive immune responses and inhibit the proinflammatory effects of the cells, including lymphocytes [27]. Foxp3 is a transcription inhibitor factor that inhibits the activity of ROR*γ*t [28, 29].

The present study used two types of interventions, electroacupuncture and an antagonist. The results showed that both interventions improved the SBP of SHRs. A previous study [30] showed that AG490 regulated Th17/Treg via inhibition of the differentiation of the Th17 cells and promotion of the differentiation of the Treg cells. AG490 promoted an increase in molecules related to Foxp3+ and the development of Treg cells [22]. The results of our study are consistent with these prior studies. Our results showed that AG490 downregulated ROR*γ*t and upregulated Foxp3 expression, decreased Th17 production and increased Treg cell production, decreased IL-6 and IL-17 secretion, and increased TGF-*β*1 and IL-10 secretion.

The present study selected acupoints ST9 and LR3 for an electroacupuncture intervention. Based on Traditional Chinese Medicine (TCM) theories, ST9 belongs to the Stomach Meridian, which has abundant *Qi and blood*. Application of acupuncture at this acupoint regulates the *Qi and blood*. LR3 is the yuan acupoint of the Liver Meridian, and regulates the activity of *Qi* to balance blood pressure. The anatomical location of ST9 is close to the carotid sinus, which regulates blood pressure [31]. Animal experiments [32–34] and clinical trials [35, 36] demonstrated that acupuncture at ST9 and LR3 significantly reduced blood pressure. Our results are consistent with these previous studies.

First, we found that electroacupuncture significantly improved the SBP of SHRs. Second, electroacupuncture effectively reduced the generation of Th17 cells and increased the generation of Treg cells. Third, electroacupuncture decreased the protein expression of ROR*γ*t, and increased the expression of Foxp3. Fourth, electroacupuncture also regulated the various cytokines secreted by the T cells and inhibited IL-6 and IL-17, and increased the secretion of TGF-*β*1 and IL-10. These results suggest that electroacupuncture improved the SBP of SHRs by regulating the balance of Th17/Treg. Another study [29] showed that TGF-*β* promoted the differentiation of Th17 cells and Treg cells. Low concentrations of TGF-*β* induced the differentiation of Th17 cells together with IL-6. Conversely, high concentrations induced the expression of Treg cells. The results of our study are consistent with the above findings. The lowest secretion of TGF-*β*1 and the highest secretion of IL-6 were found in the Model in the present study, and these cytokines jointly induced the differentiation of Th17 cells in the Model. The secretion of TGF-*β*1 was significantly higher in SHR + EA rats than Model rats, and IL-6 was significantly lower. The results also showed that the differentiation of the Treg cells in SHR + EA rats was higher than the Model.

Experiments on animals showed that the T cells and their secreted cytokines played an important role in several experimental hypertension models. Some stimulating substances, such as angiotensin II (AngII), deoxycorticosterone acetate-salt, and excessive catecholamines, resulted in the formation of Th17 cells, which penetrated into renal arteries and the perivascular areas [2], and these cells were related to the damage of target organs, such as the kidney [37]. IL-17 and IL-6 secreted by the Th17 cells promoted a renal and vascular dysfunction and damage, which resulted in the increased water-sodium retention and systemic vascular resistance and further exacerbated the development of hypertension [2, 38–40]. Previous studies [38, 41] showed that a reduction in the secretion of IL-6 and IL-17 reduced the formation of Th17 cells, the degree of atherosclerosis, and the production of proteinuria. The present study also found that electroacupuncture for SHRs downregulated the ROR*γ*t protein expression, reduced the Th17 cell differentiation, and decreased the secretion of the proinflammatory factors IL-17 and IL-6.

The adoptive transfer of Treg cells prevented hypertension induced by AngII and aldosterone [27]. Implantation of normal thymus into SHRs restored the T cell function and reduced the blood pressure [42]. A previous study [43] has found that antithymocyte serum effectively reduced the blood pressure in SHRs. Increased secretion of IL-10 and TGF-*β* had a protective effect on hypertension and regulated the endothelial function, dilated the blood vessels [44], and reduced proteinuria and renal injury in patients with hypertension [45]. Treg cells prevent hypertension via the inhibition of innate and adaptive immune responses [12, 27, 46], and these cells reduce blood pressure by releasing IL-10 [27, 47]. Treg cells also inhibit the Th17 cells polarization and the expression of IL-17 [12, 29]. Electroacupuncture treatment increased the protein expression of Foxp3, Treg cell differentiation, and the secretion of TGF-*β*1 and IL-10.

In brief, electroacupuncture inhibited Th17 cell generation and the secretion of its transcription factors and cytokines and promoted Treg cells generation and the secretion of its transcription factors and cytokines. There was an imbalance of Th17/Treg in SHRs, and electroacupuncture improved the SBP of SHRs by regulating the differentiation of Th17 cells and Treg cells. Therefore, we speculated that electroacupuncture would improve the blood pressure of SHRs by regulating the balance of Th17/Treg.

Our study had some limitations. First, only the electrical stimulation parameter of “2/15 Hz, 1 mA, each lasting for 15 minutes” was used, and no other electrical stimulation parameters were applied. Additional stimulus groups will be examined in our future studies. Second, we only examined the protein expression of Foxp3 and ROR*γ*t in kidney tissue after electroacupuncture stimulation. Further studies are required to investigate other target organ injury parameters, such as renal inflammatory injury and vascular structural changes after electroacupuncture stimulation. Third, we did not focus on the effect of JAK2/STAT3 in Th17 cells, which may be further studied in the future to reveal whether electroacupuncture played a role in Th17 cells through this pathway. Fourth, we failed to adopt a blinded method in the intervention, which made the results less valid, we need to further improve this methodology in future research.

## 5. Conclusions

In conclusion, electroacupuncture promoted the balance between Th17 and Treg and made the Th17/Treg balance axis trend towards the Treg cell lineage, which maintained the Th17/Treg balance in a steady state and improved the blood pressure of SHRs. This balance may be one of the immune mechanisms by which electroacupuncture improves blood pressure.

## Figures and Tables

**Table 1:** Location of the acupoints.

Acupoints	Anatomical locations
ST9	In the cervical triangle, at the junction of the earlobe, sternohyoid muscle and sternocleidomastoid muscle, and at the pulsation of the carotid artery

LR3	The first and second metatarsal bones on the dorsum of the foot

**Table 2:** Western blot antibodies.

Antibody	Producer
Goat anti-mouse IgG H&L	ZSGB-BIO
Goat anti-rabbit IgG H&L	ZSGB-BIO
Mouse anti-*β*-actin mAb	ZSGB-BIO
ROR anti-ROR*γ* antibody	BIOSS
Anti-FOXP3 antibody	BOSTER

**Table 3:** Comparison of SBP in rats (mmHg).

Group	Baseline	Week 1	Week 2	Week 3	Week 4
Normal	138.13 ± 8.49	139.25 ± 8.97	142.88 ± 7.16	140.38 ± 8.91	139.5 ± 7.39
Model	184.13 ± 5.08^*∗*^	193.88 ± 12.04^*∗*^	200.13 ± 8.39^*∗*^	205.75 ± 7.31^*∗*^	205.13 ± 7.77^*∗*^
SHR + AG490	181.75 ± 10.18^*∗*^	181.88 ± 10.87^*∗*^	182.88 ± 7.99^*∗#*^	186.63 ± 9.35^*∗#*^	177.63 ± 7.54^*∗#*^
SHR + EA	182.38 ± 9.75^*∗*^	189.88 ± 13.45^*∗*^	187.63 ± 11.89^*∗#*^	189.38 ± 6.7^*∗#*^	184.38 ± 10.2^*∗#*^

^*∗*^ *P* < 0.05, compared with Normal; ^#^*P* < 0.05, compared with Model.

## Data Availability

The data used to support the findings of this study are available from the corresponding author upon request.

## Supplementary Materials

Supplementary Materials Because some of the experiments we bought are ready-made kits, such as ELISA kits, the operation of these reagents is carried out according to the reagent instructions.

## References

[1] Blacher J., Levy B. I., Mourad J. J., Safar M. E., Bakris G. (2016). From epidemiological transition to modern cardiovascular epidemiology: hypertension in the 21st century. *The Lancet*.

[2] McMaster W. G., Kirabo A., Madhur M. S., Harrison D. G. (2015). Inflammation, immunity, and hypertensive end-organ damage. *Circulation Research*.

[3] Solak Y., Afsar B., Vaziri N. D. (2016). Hypertension as an autoimmune and inflammatory disease. *Hypertension Research*.

[4] Shao J., Nangaku M., Miyata T. (2003). Imbalance of T-cell subsets in angiotensin II-infused hypertensive rats with kidney injury. *Hypertension*.

[5] Kvakan H., Kleinewietfeld M., Qadri F. (2009). Regulatory T cells ameliorate angiotensin II-induced cardiac damage. *Circulation*.

[6] Littman D. R., Rudensky A. Y. (2010). Th17 and regulatory T cells in mediating and restraining inflammation. *Cell*.

[7] Diller M. L., Kudchadkar R. R., Delman K. A., Lawson D. H., Ford M. L. (2016). Balancing inflammation: the link between Th17 and regulatory T cells. *Mediators Inflammation*.

[8] Ivanov I. I., McKenzie B. S., Zhou L. (2006). The orphan nuclear receptor ROR*γ*t directs the differentiation program of proinflammatory IL-17+ T helper cells. *Cell*.

[9] Amador C. A., Barrientos V., Peña J. (2014). Spironolactone decreases DOCA-salt-induced organ damage by blocking the activation of T helper 17 and the downregulation of regulatory T lymphocytes. *Hypertension*.

[10] Sakaguchi S., Ono M., Setoguchi R. (2006). Foxp3+CD25+CD4+ natural regulatory T cells in dominant self-tolerance and autoimmune disease. *Immunological Reviews*.

[11] Schiffrin E. L. (2013). The immune system: role in hypertension. *Canadian Journal of Cardiology*.

[12] Vignali D. A. A., Collison L. W., Workman C. J. (2008). How regulatory T cells work. *Nature Reviews Immunology*.

[13] Miyazono K., Kusanagi K., Inoue H. (2001). Divergence and convergence of TGF-?/BMP signaling. *Journal of Cellular Physiology*.

[14] Wang D., Calabrese E. J., Lian B., Lin Z., Calabrese V. (2018). Hormesis as a mechanistic approach to understanding herbal treatments in traditional Chinese medicine. *Pharmacology Therapeutics*.

[15] Zhao X. F., Hu H. T., Li J. S. (2015). Is acupuncture effective for hypertension? A systematic review and meta-analysis. *PLoS One*.

[16] Li D. Z., Zhou Y., Yang Y. N. (2014). Acupuncture for essential hypertension: a meta-analysis of randomized sham-controlled clinical trials. *Evidence Based Complementary Alternative Medicine*.

[17] Liu A.-J., Li J.-H., Li H.-Q. (2015). Electroacupuncture for acute ischemic stroke: a meta-analysis of randomized controlled trials. *The American Journal of Chinese Medicine*.

[18] Li P., Pitsillides K. F., Rendig S. V., Pan H.-L., Longhurst J. C. (1998). Reversal of reflex-induced myocardial ischemia by median nerve stimulation. *Circulation*.

[19] Torres-Rosas R., Yehia G., Peña G. (2014). Dopamine mediates vagal modulation of the immune system by electroacupuncture. *Nature Medicine*.

[20] Liu Y.-M., Liu X.-J., Bai S.-S. (2010). The effect of electroacupuncture on T cell responses in rats with experimental autoimmune encephalitis. *Journal of Neuroimmunology*.

[21] Hua X. B., Zhou H. L. (1991). Draft of atlas of acupuncture points for rat. *Laboratory Animals and Animal Experiments*.

[22] Davoodi-Semiromi A., Wasserfall C. H., Xia C. Q. (2012). The tyrphostin agent AG490 prevents and reverses type 1 diabetes in NOD mice. *PLoS One*.

[23] Caceres-Cortes J. (2008). A potent anti-carcinoma and anti-acute myeloblastic leukemia agent, AG490. *Anti-Cancer Agents in Medicinal Chemistry*.

[24] Huang C., Yang G., Jiang T., Huang K., Cao J., Qiu Z. (2010). Effects of IL-6 and AG490 on regulation of Stat3 signaling pathway and invasion of human pancreatic cancer cells in vitro. *Journal of Experimental & Clinical Cancer Research*.

[25] Eriksen K., Kaltoft K., Mikkelsen G. (2001). Constitutive STAT3-activation in Sezary syndrome: tyrphostin AG490 inhibits STAT3-activation, interleukin-2 receptor expression and growth of leukemic sezary cells. *Leukemia*.

[26] Kato K., Wakai J., Ozawa K., Sekiguchi M., Katahira K. (2016). Different sensitivity to the suppressive effects of isoflurane anesthesia on cardiorespiratory function in SHR/Izm, WKY/Izm, and Crl:CD (SD) rats. *Experimental Animals*.

[27] Barhoumi T., Kasal D. A., Li M. W. (2011). T regulatory lymphocytes prevent angiotensin II-induced hypertension and vascular injury. *Hypertension*.

[28] Ichiyama K., Yoshida H., Wakabayashi Y. (2008). Foxp3 inhibits ROR*γ*t-mediatedIL-17AmRNA transcription through direct interaction with ROR*γ*t. *Journal of Biological Chemistry*.

[29] Zhou L., Lopes J. E., Chong M. M. W. (2008). TGF-*β*-induced Foxp3 inhibits TH17 cell differentiation by antagonizing ROR*γ*t function. *Nature*.

[30] Park J.-S., Lee J., Lim M.-A. (2014). JAK2-STAT3 blockade by AG490 suppresses autoimmune arthritis in mice via reciprocal regulation of regulatory T cells and Th17 cells. *The Journal of Immunology*.

[31] Zhang L., Lai H., Li L. (2019). Effects of acupuncture with needle manipulation at different frequencies for patients with hypertension: result of a 24- week clinical observation. *Complementary Therapies in Medicine*.

[32] Cao X., Lu S., Ohara H. (2015). Beneficial and adverse effects of electro-acupuncture assessed in the canine chronic atrio-ventricular block model having severe hypertension and chronic heart failure. *Acupuncture & Electro-Therapeutics Research*.

[33] Lee S.-N. C., Ho T. J., Shibu M. A. (2016). Protective effects of electroacupuncture at LR3 on cardiac hypertrophy and apoptosis in hypertensive rats. *Acupuncture in Medicine*.

[34] Lai X., Wang J., Nabar N. R. (2012). Proteomic response to acupuncture treatment in spontaneously hypertensive rats. *PLoS One*.

[35] Yang Y., Zhou G. T. (2010). A comparative study of the acupuncture formula in the treatment of hypertension. *Acta Chinese Medicine Pharmacology*.

[36] Wei Y., Sun Z. R., Kou J. Y., Guo Y. (2006). Clinical observation of 120 cases with hypertension treated with acupuncture at Renying point. *Journal of Clinical Acupuncture and Moxibustion*.

[37] Harrison D. G., Marvar P. J., Titze J. M. (2012). Vascular inflammatory cells in hypertension. *Frontiers in Physiology*.

[38] Lee D. L., Sturgis L. C., Labazi H. (2006). Angiotensin II hypertension is attenuated in interleukin-6 knockout mice. *American Journal of Physiology Heart and Circulatory Physiology*.

[39] Luther J. M., Gainer J. V., Murphey L. J. (2006). Angiotensin II induces interleukin-6 in humans through a mineralocorticoid receptor-dependent mechanism. *Hypertension*.

[40] Madhur M. S., Lob H. E., McCann L. A. (2010). Interleukin 17 promotes angiotensin II-induced hypertension and vascular dysfunction. *Hypertension*.

[41] Wu J., Thabet S. R., Kirabo A. (2014). Inflammation and mechanical stretch promote aortic stiffening in hypertension through activation of p38 mitogen-activated protein kinase. *Circulation Research*.

[42] Ba D., Takeichi N., Kodama T., Kobayashi H (1982). Restoration of T cell depression and suppression of blood pressure in spontaneously hypertensive rats (SHR) by thymus grafts or thymus extracts. *Journal of Immunology (Baltimore, Md.: 1950)*.

[43] Bendich A., Belisle E. H., Strausser H. R. (1981). Immune system modulation and its effect on the blood pressure of the spontaneously hypertensive male and female rat. *Biochemical and Biophysical Research Communications*.

[44] Didion S. P., Kinzenbaw D. A., Schrader L. I., Chu Y., Faraci F. M. (2009). Endogenous interleukin-10 inhibits angiotensin II-induced vascular dysfunction. *Hypertension*.

[45] Lai Z., Kalkunte S., Sharma S. (2011). A critical role of interleukin-10 in modulating hypoxia-induced preeclampsia-like disease in mice. *Hypertension*.

[46] Schiffrin E. L. (2014). Immune mechanisms in hypertension and vascular injury. *Clinical Science*.

[47] Kasal D. A., Barhoumi T., Li M. W. (2012). T regulatory lymphocytes prevent aldosterone-induced vascular injury. *Hypertension*.

