# Supplementary material for: Electroacupuncture Improves Blood Pressure in SHRs by Regulating the Immune Balance between Th17 and Treg
Source: Evid Based Complement Alternat Med. 2020 Jul 7;2020:5375981. doi: 10.1155/2020/5375981 (PMC7366219; doi:10.1155/2020/5375981)
Supplement: Supplementary Materials — Because some of the experiments we bought are ready-made kits, such as ELISA kits, the operation of these reagents is carried out according to the reagent instructions. [file 5375981.f1.pdf]

Experiment	Material and Reagent	Producer	Cat#:	Description
Western Blot	BCA Protein Assay Kit	Boster	AR0146	protein determination: Ensure that the total amount of each protein sample is the same
	RIPA Lysis buffer	Solarbio	R0020	lysate of cells and tissues to obtain protein samples
	Loading buffer	Solarbio	P1015	It serves as an indicator and makes the sample sink into the sample hole
	Mouse Anti-β actin mAb	ZSGB-BIO.CO.Ltd	TA-09	reference protein
	ROR Anti-RORγ antibody	BIOSS	BS-6217R	first antibody: The protein to be studied combines with a first antibody to form an antigen antibody complex
	Anti-FOXP3 Antibody	Boster	BA2032-2	
	IgG(H+L) HRP Peroxidase-Conjugated Goat anti-Mouse IgG	ZSGB-BIO.CO.Ltd	ZB2305	second antibody: The antibody complex formed by the combination of labeled second antibodies and first antibody can indicate the location of the first antibody, that is, the location of the protein to be studied
	IgG(H+L) HRP Peroxidase-Conjugated Goat anti-Rabbit IgG	ZSGB-BIO.CO.Ltd	ZB2301	
Flow cytometry	CD4 -AF488	Biolegend	201511	antibody: Binding to specific surface or intracellular
	FoxP3- APC	Biolegend	17-5773-82	
	CD25- PE	Biolegend	202105	
	CD3- APC	Biolegend	201414	

	CD8a- PerCP	Biolegend	201712	markers of target cells
	IL-17A- PE	Biolegend	12-7177-81	
	RBC Lysis Buffer	CWBIO	CW0613S	Red Blood Cell Lysis Buffer: lyse red blood cells. It cannot damage the nucleated cells, but also remove the red blood cells
	IntraSure™Kit	BD Pharmingen	641776	fixing and permeabilizing of cells to allow staining of intracellular targets
	LEUKO ACTVTN CKTL WITH GLGPLG	BD Pharmingen	349202	inducing cytokine-producing cells
	Transcription Factor Buffer Set	BD Pharmingen	562574	fixing and permeabilizing cells prior to flow cytometric analysis of cells that express specific intracytoplasmic and intranuclear proteins
	HyClone™ RPMI Medium Modified	HyClone	SH30809.01	culture medium
	PBS	HyClone	SH30256.01	Wash buffer
ELISA	TGF-β1ELISA	BOSTER	EK0514	enzyme linked immunosorbent assay kit: Double antibody sandwich method
	IL-10ELISA	BOSTER	EK0418	
	IL-6ELISA	BOSTER	EK0412	
	IL-17AELISA	DAKEWE	1311702	
